# Objective assessment of accommodation with an accommodative intraocular lens: Development of methodology and case report

**DOI:** 10.1016/j.zemedi.2026.03.005

**Published:** 2026-03-10

**Authors:** Timo Eppig, Violetta Müller, Manuel Seer, Alisa Bölke, Arthur Meßner, Michiel C. Rombach, Eckhard Becker, Jens Schrecker

**Affiliations:** a AMIPLANT GmbH, Haidling 1, Schnaittach, 91220, Germany; b Institut für Experimentelle Ophthalmologie, Universität des Saarlandes, Kirrberger Str. 100, Homburg/Saar, 66424, Germany; c Augentagesklinik Oranienburg MVZ GmbH, Breite Str. 7, Oranienburg, 16515, Germany; d Akkolens Clinical b.v., Polakweg 10-12, Rijswijk, 2288 GG, The Netherlands; e Klinik für Augenheilkunde, Rudolf Virchow Klinikum, Virchowstr. 18, Glauchau, 08371, Germany

**Keywords:** Cataract, Intraocular lens, Accommodation measurement

## Abstract

**Purpose:** To determine the objectively measurable amplitude of accommodation after implantation of an accommodating intraocular lens (IOL).

**Methods:**

We used an open field autorefractor based on near infrared retinoscopy (PowerRef3, Plusoptix GmbH, Nuremberg, Germany) to determine the change in refraction, pupil diameter and gaze with decreasing distance of the visual stimulus. We compared the signal curves between a young healthy, a presbyopic and a pseudophakic patient as well as three patients implanted with the accommodative IOL Lumina (AMIPLANT GmbH, Schnaittach, Germany).

**Results:**

The refraction, pupil, and gaze curves exhibit the behavior that are expected from theory. The accommodative IOL shows similar curves as the young phakic person, showing that there is a dynamic change in refraction. This is supported by the three-year follow-up data of a patient providing an uncorrected binocular far VA of 1.0 and near visual acuity of 0.8.

**Conclusion:**

The PowerRef3 is capable of measuring the accommodative amplitude of an accommodative IOL. The Lumina shows a accommodative amplitude of up to 2 D and excellent far and near vision in a patient three years after implantation.

## Highlights

• We demonstrate a method how to objectively investigate accommodation in phakic and pseudophakic patients.
• We present exmaples of accommodative response curves of phakic, presbyopic and pseudophakic patients.
• We report about a single case’s visual performance results with a follow-up of 3 years.
• The accommodative intraocular lens shows up to 2 D of accommodation in that patient 3 years after cataract surgery.

## 1 Introduction

Modern cataract surgery not only claims for pure restoration of visual function, patients more and more desire full spectacle independence after surgery. Previous concepts for the correction of presbyopia in the context of cataract surgery are usually based on dividing the incident light into two or more focal points for the distance, intermediate and near range. This concept is implemented in conventional multifocal IOLs [1]. Newly developed extended depth of focus (EDoF) concepts increase the depth of focus by either reducing the effective aperture or inducing an intentional amount of aberrations. However, the distribution of the amount of light also causes disadvantages, such as a reduction in contrast vision and frequent occurrence of photic phenomena such as glare or halos [2], [3], [4], [5]. These phenomena are already significantly reduced in newer Extended Depth of Focus (EDoF) lenses based on non-diffractive principles and enhanced monofocal lenses [6], [7], which could be placed in the gap between monofocal and EDoF IOLs [8]. However, these types of IOLs (intraocular lenses) are only optimized for a limited intermediate range up to approx. 1.75 D or less in enhanced monofocal lenses. Higher spectacle independence could potentially be achieved by expanding the options for treatment using mix-and-match approaches combining monovision and or trifocal lenses with EDoF lenses [9].

**Fig. 1:**
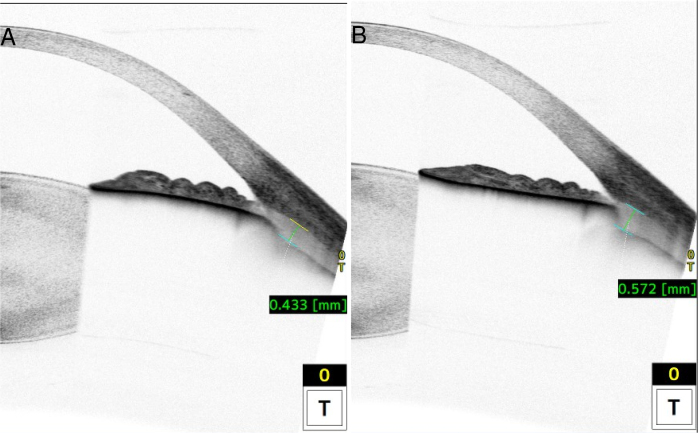
Anterior Segment optical coherence tomographer (OCT) scan (CASIA2, Tomey Corp., Nagoya, Japan) of an eye in (A) relaxed state with 433μm muscle thickness, and (B) under 3 D of accommodation with 572μm.

Stachs et al. already showed in 2002 that the motility of the ciliary muscle is still present in the aged, presbyopic eye [10]. Fig. 1 shows an optical coherence tomographer (OCT) scan of an eye of a pre-presbyope 46 year old in relaxed state and under 3 D of accommodation. The change in the shape of the ciliary muscle is clearly visible, the diameter of the ciliary sulcus decreased by approx 0.3 mm as measured by the method previously described [11]. This is the principal prerequisite for any accommodative concept.

However, most of these IOL-concepts were positioned inside the capsular bag as it is the case in conventional cataract surgery. There were several principles described in literature: The first type of lenses worked according to the translation principle, i.e. the plane of action of the optics is shifted forward for accommodation. Representatives of this type included the 1CU (HumanOptics AG, Erlangen, Germany) and the Crystalens HD (Bausch+Lomb, Rochester, USA) [12], [13]. In addition, multiple optics based on the translation principle were also investigated, such as the Synchrony IOL (Visiogen, Irvine, USA) [13], [14], [15]. The lenses in the other group were based on a change in the radius curvature of the IOL or in the refractive index of the lens material, similar to the natural lens. This also includes lens refilling, in which an attempt was made to refill the lens capsule with a gel-like flexible lens [16], [17], [18]. Some of these concepts even seemed to work successfully for a short time in the postoperative course. Currently, there are few capsular bag based accommodative lenses in the focus of clinical research. However, postoperative capsular fibrosis is a significant problem that leads to both stiffening and shrinkage of the capsular bag and thus to the restriction of mobility [19], [20]. Latest models such as the Juvene (Lensgen, Irvine, USA) and the OmniVu (ATIA Vision Inc., Campbell, USA) use a modular cage system with a posterior lens and a secondary lens that provides the required power for the eye [21]. Both are currently investigated in clinical trials. These cage systems may act like a thick capsular tension ring which have been shown to have a positive effect on posterior capsule opacification [22], [23], [24], [25]. Despite the fact that the visual impairment caused by posterior capsule opactification (PCO), a common side effect of cataract surgery, could easily be solved by performing Nd:YAG capsulotomy, one has to keep in mind that the reduction in transparency is caused by fibrosis, which compromises the elasticity of the capsular bag and, therefore, the transmission ciliary muscle induced force to the IOL [26]. In order to achieve direct force transmission from the ciliary body to the lens, a direct coupling of the haptics to the ciliary muscle without the involvement of the capsular bag seems to be necessary.

**Fig. 2:**
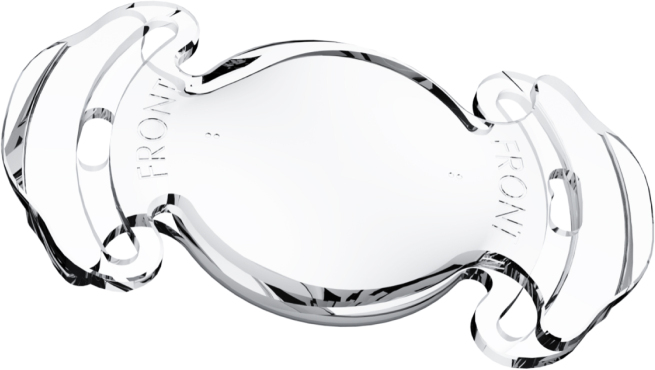
3D rendering of the Lumina potentially accommodative intraocular lens with a “‘FRONT”’ engraving.

**Fig. 3:**
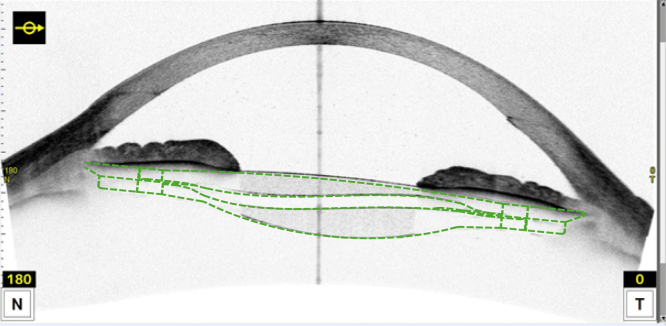
Anterior Segment optical coherence tomographer (OCT) scan (CASIA2, Tomey Corp., Nagoya, Japan) of an eye with a Lumina implant. The dashed overlay lines represent a CAD model of the lens.

**Fig. 4:**
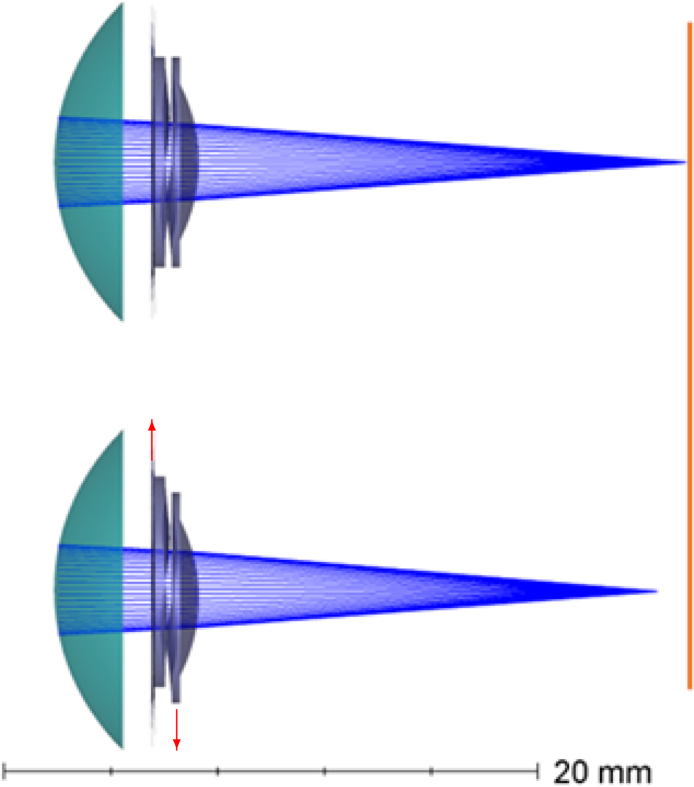
Optical simulation of the Lumina IOL. The contralateral movement of the two lens components induces an increase in optical power of 3–4 D at spectacle plane.

**Fig. 5:**
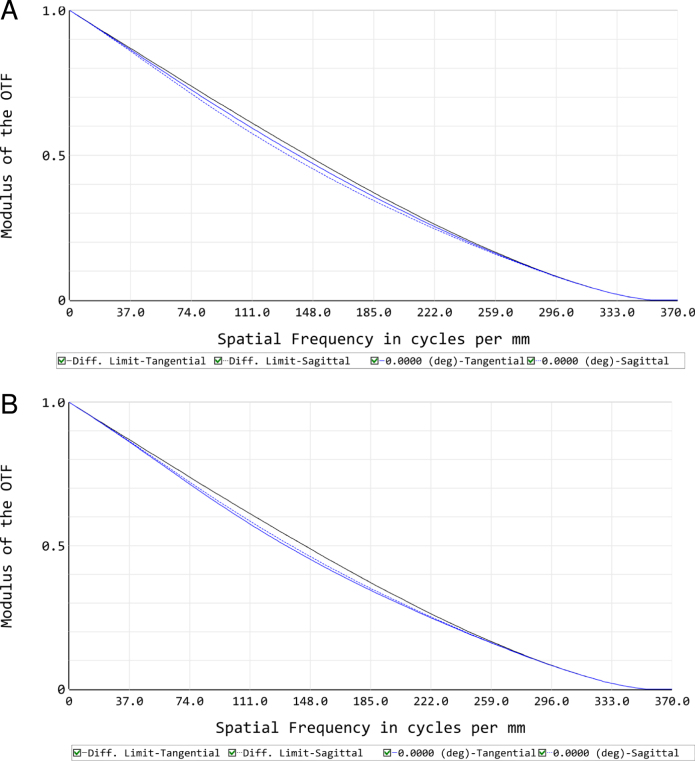
The modulation transfer function is nearly equal and diffraction limited in relaxed (A) and accommodated (B) state.

The Lumina IOL (Akkolens Clinical b.v., Rijswijk, The Netherlands; manufactured by AMIPLANT GmbH, Schnaittach, Germany) goes beyond these approaches with its principle of variable focusing. The Lumina (Fig. 2) is based on the Alvarez lens, which allows variable change in optical power in order to provide pseudophakic accommodation. In this case, two lenses with free-form geometry move in opposite directions to each other [27]. The Alvarez principle was described as early as 1964 and has been implemented ever since in a variety of optical systems, such as endoscopes [28]. The idea of using this concept for intraocular lenses is also not entirely new. For example, the mechatronic accommodation system, which was developed by the Karlsruhe Institute of Technology in cooperation with Rostock University, is also based on this optical principle [29]. The Lumina, however, is a purely passive implant made from hydrophilic acrylic polymer (n = 1.46). Unlike conventional intraocular lenses, the Lumina is placed into the ciliary sulcus as shown in Fig. 3, where it is driven directly by the centripetal movement of the ciliary muscle. The two lens components are connected to each other via the haptics. An intentional space of 0.36 mm between the two lens components, which fills with aqueous humor, prevents the lenses from adhesion to each other. The haptics are designed as a solid-state spring and transmit the force of the ciliary muscle to the two lenses, which then move in opposite directions changing the total refractive power of the lens (Fig. 4). The optical quality (modulation transfer function) in the model eye is nearly independent from this change in optical power (Fig. 5).

The ciliary sulcus seems to be an ideal implantation site for this purpose. Although this is not considered the standard implantation site of first choice for an intraocular lens, it allows the IOL to be securely positioned but some limitations may apply such as a lens design that prevents iris chafing [30]. Since the anatomical position of the ciliary sulcus can also be used to predict the postoperative lens position relatively accurately, no additional formulas or lens constants are required for the calculation of the lens refractive value. In order to achieve an ideal mechanical coupling of the lens to the ciliary mass, the diameter of the ciliary muscle needs to be measured accurately. The former gold standard for this is ultrasound biomicroscopy. However, this is a rather time consuming, burdensome method with limited precision. An alternative method to determine the size of the ciliary sulcus is the use of a dedicated anterior segment optical coherence tomographer (OCT) as described previously [11]. Although the light of most ophthalmic OCT devices is unable to penetrate the iris pigment, devices operating at mid infrared wavelengths of 1310 nm obviously provide information from this region. Additional image acquisition strategies such as eye-tracking and averaging to reduce noise allow to increase the contrast for more accurate measurement of the sulcus-to-sulcus diameter.

Currently, the Lumina is available in a continuous refractive power range from 10 to 30 diopters and in a size range from 10.85 to 12.50 mm (theoretically 0.01 increments). The lens is manufactured according to the sulcus diameter with an accuracy of ±50 μm. The lens’ optic is aspheric to compensate for corneal spherical aberration [31], a toric variant is currently not available. The optic calculation is performed via ray-tracing because conventional thin- and thick-lens-formulae cannot be applied to the dual optic lens [32], [33]. The lens is made of hydrophilic acrylate with 26% water content and is available with and without a blue light filter. The Lumina can be implanted as part of a standardized cataract surgery with an incision size of 2.2 mm to 3.0 mm (depending on the size of the injector and the refractive power of the lens). Similarly to the routine with phakic IOLs, implantation in the sulcus takes place in a 0°–180° position, as the diameter of the ciliary sulcus can only be reliably measured in the horizontal axis without manually opening the eyelids. Preliminary study results with previous investigational models of the Lumina showed promising potential regarding refractive outcome and spectacle independence [34], [35], [36], [37]. The phase III results with 25 bilateral patients showed a uncorrected distance visual acuity of 0.06 ± 0.15 logMAR and an uncorrected near visual acuity of 0.27 ± 0.15 logMAR. Eighty-seven percent of the patients reported only mild or no problems in uncorrected near vision. However, objective accommodation was only as low as 0.65 ± 0.69 D. In addition, photic phenomena occur on a scale comparable to those of a monofocal lens, there were no reports about halo and glare [34], [35], [36], [37]. The Lumina bears a CE mark since 2021 and has been implanted in Spain, Germany, the Netherlands, and Belgium as part of a multicenter post-market clinical follow-up.

The purpose of this research is to develop a methodology to determine dynamic accommodation ability with an accommodative IOL.

## 2 Patients & methods

The study is being conducted at 11 clinical sites throughout Europe (4 in Germany, 4 in the Netherlands, 2 in Spain, and 1 in Belgium). Each clinical shall recruit up to 20 patients (40 eyes). Permission from the local ethical review boards was granted and the study adheres to the Tenets of the Declaration of Helsinki. The study patients are to be recruited from the outpatient departments of the clinics. The patients undergo full preoperative ophthalmological examination including fundus examination, IOLMaster (IOLMaster 700, Carl Zeiss Meditec, Jena, Germany), anterior segment OCT (CASIA2, Tomey Corp., Nagoya, Japan) with a customized scan protocol mentioned previously [11]. The power and size of the Lumina was then calculated by the manufacturer from the OCT data using ray tracing. Postoperatively, the patients undergo 7 follow-ups at 1 day, 1–2 weeks, 1–3 months. 3–6 months, 1 year, 2 years and 3 years after surgery. The postoperative examinations include full ophthalmological examination, visual acuity (far, intermediate, near), defocus curves, objective accommodation assessment, and the completion of the Near Activitiy Visual Questionnaire (NAVQ) [38] and the Quality of Vision (QoV) questionnaire [39].

### 2.1 Accommodation measurement

**Fig. 6:**
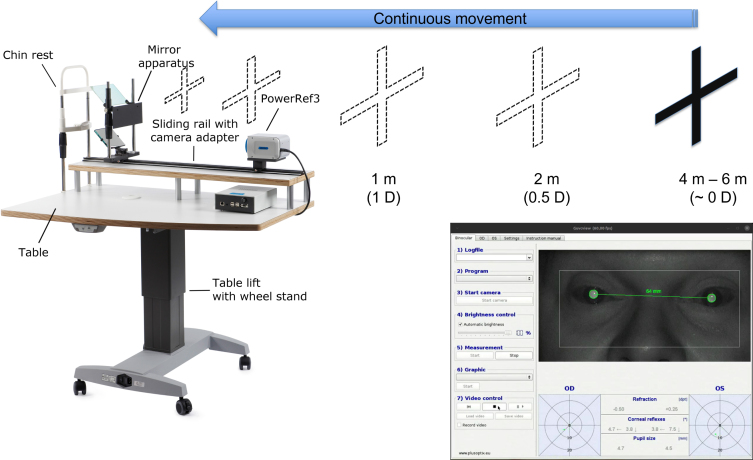
Sketch of the objective accommodation assessment method. The target is moved continuously from far to near distance while the PowerRef3 is recording the refraction, pupil size and gaze angles of both eyes.

Measurement of objective accommodation was performed using the PowerRef3 device (Plusoptix GmbH, Nuremberg, Germany), which uses dynamic videoretinoscopy to record changes in refraction, pupil diameter and gaze angles of both eyes simultaneously (Fig. 6). The patient’s head was placed into the chin rest of the device and a ETDRS chart was placed at a distance of 6 m. The patient was then asked to fixate the target, while the measurement was started. Then, during recording, the target was moved from far distance down to approximately 30 cm distance from the patient’s eyes (Fig. 6) without controlled target speed. The patient was asked to change focus to the smallest legible line of the chart to maintain a permanent visual challenge for the eye and therefore a stimulus for accommodation. In order to demonstrate the ability of the device to detect accommodation, we recruited 3 volunteers, who solely underwent a PowerRef measurement: one 27 year old myopic female, one 58 year old presbyope male and one pseudophakic person with monofocal intraocular lenses (Tecnis monofocal). Since there was no pragmatic option for recording the stimulus distance in alignment with the PowerRef3, we used the duration of the measurement as surrogate. In addition, additional measurements were recorded at discrete target distances.

### 2.2 Defocus curves

The defocus curves were measured with a trial frame correction. For monofocal results the eye not under test was occluded with a ground cover disc. Distance correction was applied and considered as defocus level 0. Then the image plane was defocused by using a plus lens of ＋2.0 D in addition to the distance correction and the visual acuity was noted. The visual acuity was then noted for each step modifying the defocus by −0.5 D until a defocus level of −5.0 D was achieved. The visual acuity was documented for each defocus level in the data sheet as a function of defocus level.

## 3 Results

**Fig. 7:**
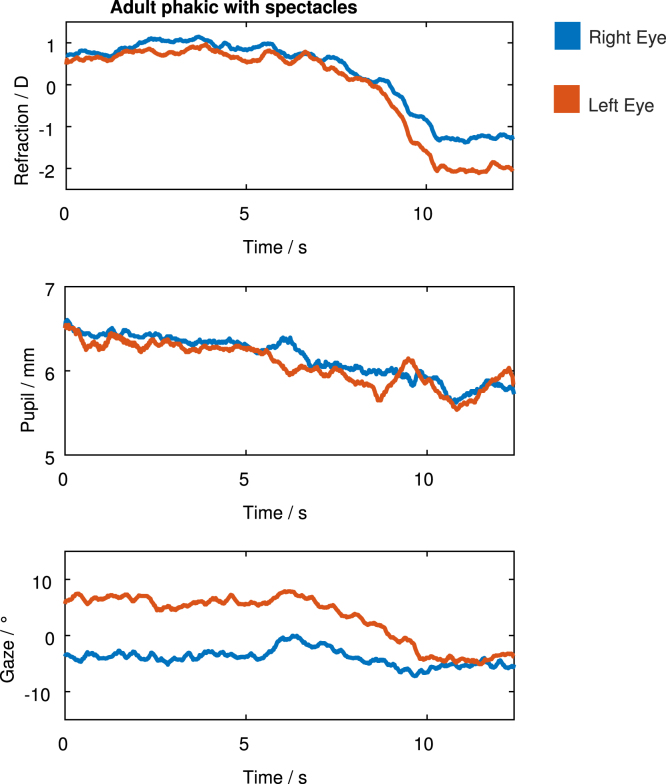
Accommodation measurement of the young phakic person (27 years old female). The change in refractive power, pupil constriction and gaze conversion are perfectly in line.

**Fig. 8:**
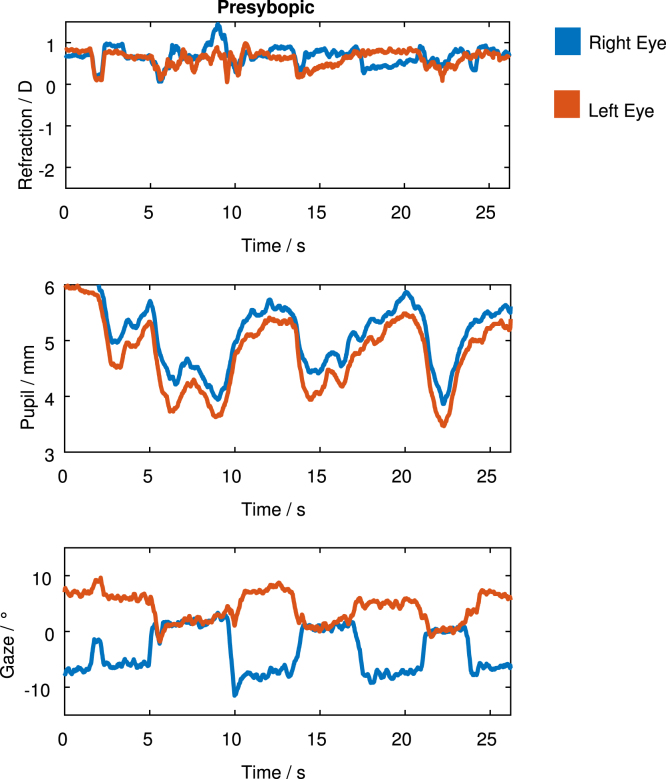
Accommodation measurement of the presbyopic person (58 years old male). Here, we used an alternating far-near stimulus to demonstrate the non existent accommodation ability. The pupil and gaze curves reveal that the test person was fixating the target but the lens could not maintain the refractive power.

**Fig. 9:**
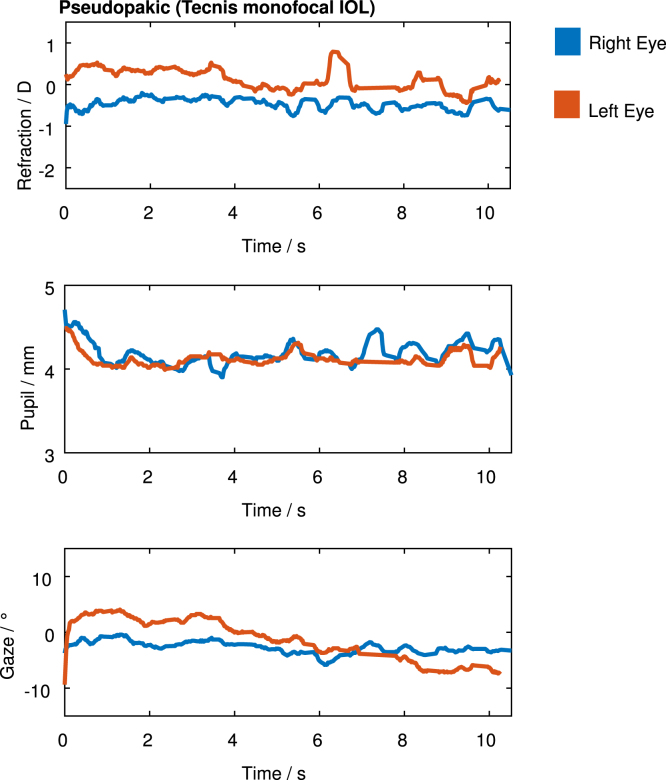
Accommodation measurement of the pseudophakic person. There is no change in refraction, which is correlating with the non existent pupil decrease. However, the eyes maintain convergence and fusion, which demonstrates that the person fixated the target.

The young phakic person Fig. 7 shows a nearly static refraction during the first 5 s with a clear shift to myopia with decreasing stimulus distance. During the entire measurement the pupil diameter is continuously decreasing increasing depth of focus. During the first 5 s, when there is no change in refraction, the eyes are parallel. With decreasing stimulus distance, the eyes start converging and fusion is attained at the maximum of accommodation after approx. 11 s. The presbyopic person, however, underwent a longer exam with repeated alternating far-near stimuli (Fig. 8) demonstrating that the person maintained convergence and fusion along with pupil constriction but there was no change in refractive power, because of the loss of crystalline lens elasticity as the primary cause for presbyopia [40]. The pseudophakic person, as excpected, showed no modulation, neither for refraction nor for pupil size. Only the gaze movement reveals that the patient tried to follow the stimulus (Fig. 9).

### 3.1 Case 1

**Fig. 10:**
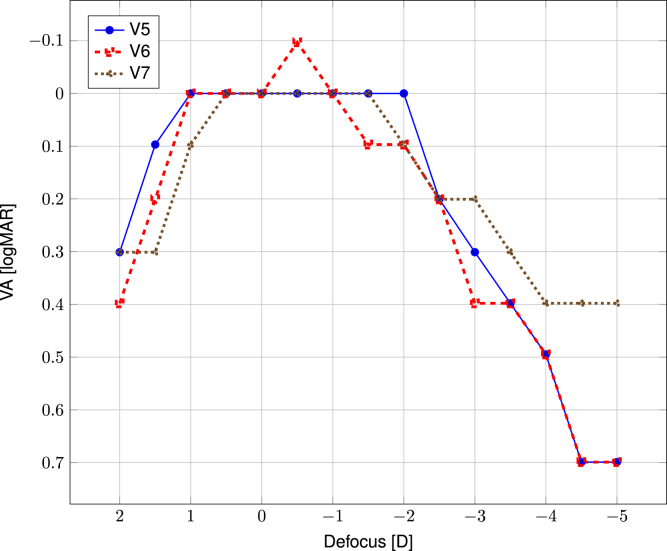
Binocular defocus curve for the 1-year (V5), 2-year (V6), and final 3-year (V7) visit.

**Fig. 11:**
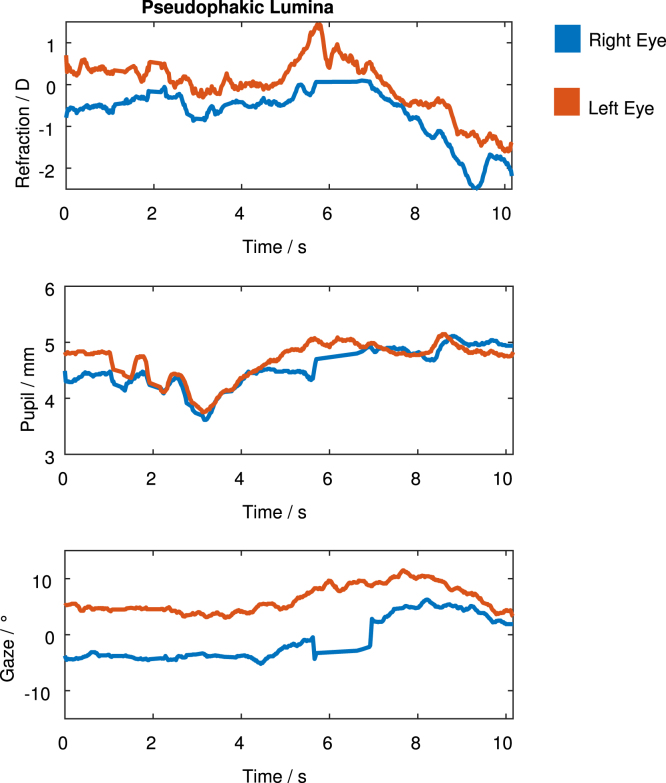
Accommodation measurement of a 46-year old Lumina patient at the 3 year follow-up. The refraction curve reveals an accommodative amplitude of up to 2 D. The eyes maintain convergence and fusion, which demonstrates that the person fixated the target.

The first implantation in Germany took place at the Rudolf Virchow Clinic in Glauchau in the summer of 2022. The 46-year-old female presented with decreased vision due to an advanced cataract. Rosacea was present as a concomitant disease. The patient had learned about the lens from research materials and explicitly wanted the Lumina to be implanted due to her drug-related cataract induced by corticosteroid therapy 20 years ago. Preoperative characteristics are shown in Table 1. Measurement of the ciliary sulcus was performed according to the protocol published previously using 3 slices at 0, 30, and 150 degrees [11]. Power and diameter of the Lumina IOLs calculated for both eyes are listed in Table 1. The uneventful implantations took place in July (OD) and November 2022 (OS). The Lumina was inserted through a superior tunnel with an injector with a 2.2 mm nozzle (AccuJect 2.2-1P; Medicel AG, Switzerland). After the procedure, follow-up examinations were carried out on the 1st postoperative day, after 1–2 weeks, as well as after 1–2 months, 4–6 months, 11–14 months (1 year), 2, and 3 years. A Nd:YAG capsulotomy was performed on the right eye 5 weeks after implantation due to a significantly reduced visual acuity. Visual acuity for the near and intermediate range was tested with ETDRS charts at distances of 40 cm and 60 cm. Near distance visual function was additionally assessed by NAVQ, photopic symptoms were assessed by the QoV questionnaire. The monocular and binocular results are shown in Table 1, Table 2, respectively. The defocus curves are shown in Fig. 10 . The NAVQ score after 3 years was 1 out of 30, the overall grading was very satisfied (1). In the QoV, the patient noted to perceive mild glare, sometimes and full satisfaction with the result as well as recommendation of the Lumina to family and friends. At the final exam, we used the PowerRef3 to determine the objective accommodation amplitude, as shown in Fig. 11. The curves reveal that an accommodative power shift of up to 2 D in a single measurement, while the pupil diameter increased. This opposite effect was caused by the dark room in which the measurement was performed, which was required to maintain the pupil diameter above 4 mm so that the PowerRef3 could analyze the signal. At that time, mydriatic medication was not used, because typical anti-muscarinic mydriatic agents affect the ciliary body. Repeat measurements were performed, but a statistical evaluation seemed not feasible due to the lacking x-axis reference (uncontrolled target speed instead of discrete distance signal).

**Table 1:** Postoperative visual results throughout the follow-up.

Visit	Time postOP	Parameter	OD	OS
V1	1 d	UDVA/DCVA [logMAR]	0.3/0.2	0.2/0.0
		SPH/CYL [D] × Axis	0.0 −0.5 × 120	−1.0
		IOP [mmHg]	14	12

V2	2 w	UDVA/DCVA [logMAR]	0.1/0.0	0.0/0.0
		SPH/CYL [D] × Axis	−0.25 −0.25 × 125	−0.25 −0.5 × 87
		IOP [mmHg]	12	11

V3	1–2 m	UDVA/DCVA [logMAR]	0.2/−0.1	0.0/−0.1
		SPH/CYL [D] × Axis	−0.5 −0.25 × 125	0.0 −0.5 × 115
		IOP [mmHg]	17	21

V4	4–6 m	UDVA/CDVA [logMAR]	0.0/−0.1	0.0/0.0
		SPH/CYL [D] × Axis	0.0 −0.5 × 120	0.0
		UIVA/DCIVA	0.2/0.3	0.0/0.0
		UNVA/DCNVA [logMAR]	0.4/0.5	0.2/0.2
		IOP [mmHg]	14	16

V5	11–14 m	UDVA/CDVA [logMAR]	0.0/0.0	0.0/0.0
		SPH/CYL [D] × Axis	−0.25 −0.25 × 115	−0.25 −0.25 × 99
		UIVA/DCIVA	0.0/0.0	0.1/0.0
		UNVA/DCNVA [logMAR]	0.1/0.1	0.5/0.2
		IOP [mmHg]	18	19

V6	2 y	UDVA/CDVA [logMAR]	0.0/−0.1	0.0/−0.1
		SPH/CYL [D] × Axis	0.0 −0.25 × 125	0.0 −0.5 × 111
		UIVA/DCIVA	0.0/0.0	−0.1/−0.1
		UNVA/DCNVA [logMAR]	0.0/0.1	0.0/0.1
		IOP [mmHg]	18	17

V7	3 y	UDVA/CDVA [logMAR]	0.0/0.0	0.0/0.0
		SPH/CYL [D] × Axis	0.0	−0.25 −0.25 × 105
		UIVA/DCIVA	0.0/0.0	0.0/0.0
		UNVA/DCNVA [logMAR]	0.2/0.2	0.1/0.2
		IOP [mmHg]	18	18
		ECD [cells/mm${}^{2}$]	2447	2519

SPH Sphere of refraction, CYL Cylinder of refraction, IOP Intraocular pressure, ECD Endothelial cell density, UDVA Uncorrected distance visual acuity, DCVA Distance corrected visual acuity, UIVA Uncorrected intermediate visual acuity, DCIVA distance corrected intermediate visual acuity, UNVA Uncorrected near visual acuity, DCNVA Distance corrected near visual acuity.

**Table 2:** Binocular visual acuity after 1,2, and 3 years.

Visit	Acuity measure [logMAR]			
OD: V5–V6/OS: V5	UDVA	0.0	CDVA	0.0
	UIVA	0.0	DCIVA	0.0
	UNVA	0.2	DCNVA	0.1

V6	UDVA	0.0	CDVA	−0.1
	UIVA	−0.1	DCIVA	−0.1
	UNVA	0.0	DCNVA	0.1

V7	UDVA	0.0	CDVA	0.0
	UIVA	−0.1	DCIVA	−0.1
	UNVA	0.1	DCNVA	0.1

UDVA Uncorrected distance visual acuity, DCVA Distance corrected visual acuity, UIVA Uncorrected intermediate visual acuity, DCIVA distance corrected intermediate visual acuity, UNVA Uncorrected near visual acuity, DCNVA Distance corrected near visual acuity.

### 3.2 Case 2

**Fig. 12:**
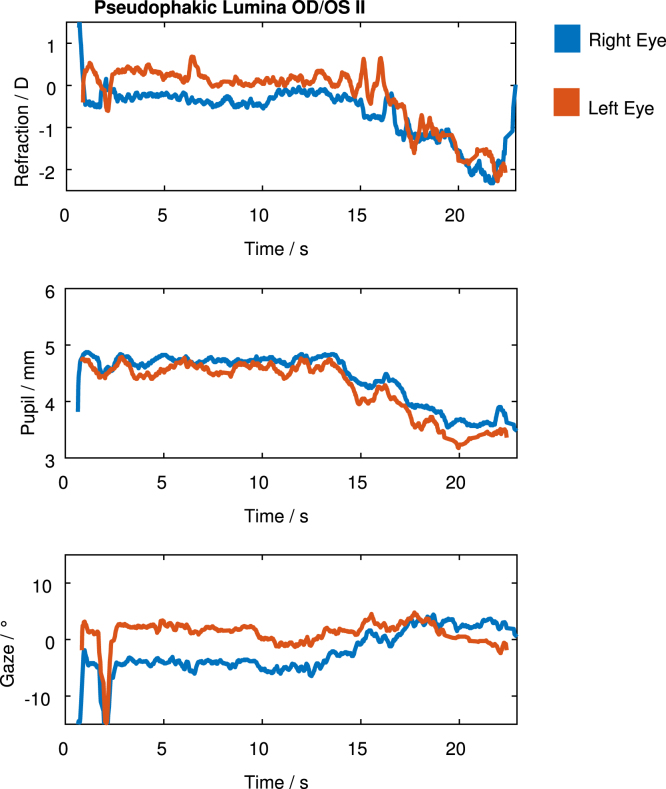
Accommodation measurement of a 70-year old Lumina patient at the 5 weeks follow-up. The refraction curve reveals an accommodative amplitude of up to 2 D. The eyes maintain convergence and fusion, which demonstrates that the person fixated the target.

A 70-year old male underwent bilateral cataract surgery with implantation of a Lumina IOL in 2025. The patient was followed up until V3 (1–2 months). Postoperative refraction at V3 was plano in both eyes. UDVA was 0.2 logMAR OD and 0.0 logMAR OS. The objective accommodation test shows significant amount of accommodation (Fig. 12) of up to 2 D at 5 weeks after surgery.

### 3.3 Case 3

**Fig. 13:**
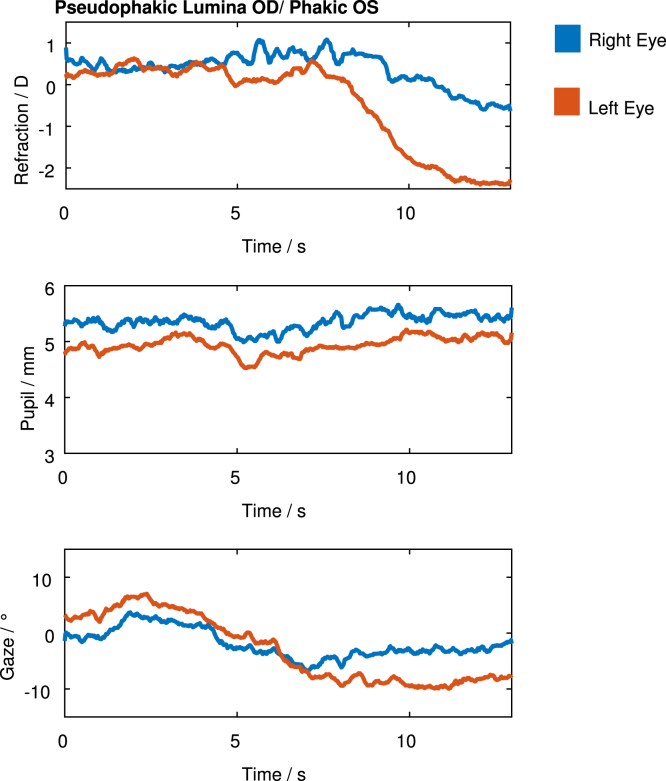
Accommodation measurement of a 42-year old patient with monocular implantation of a Lumina IOL. The curves demonstrate how both eyes show accommodation, while the phakic eye shows a significantly larger amplitude than the pseudophakic eye.

A 42-year old male underwent unilateral cataract surgery with implantation of a Lumina IOL in the right eye in 2024. Five weeks after surgery the refraction was sph ＋1.5 D cyl −0.75 D × 155 with an UDVA of 0.2 and a CDVA of 0.0 logMAR. The accommodation curve recorded at 6 weeks after surgery (Fig. 13) shows the synchronized accommodation between the pseudophakic and the phakic eye. One year after surgery, refraction was sph ＋0.75 D cyl −0.75 D × 143. UDVA and CDVA were 0.0 logMAR, DCNVA was 0.2 logMAR. The patient reported to be fully satisfied with the result.

## 4 Discussion

Restoration of accommodation has been the ultimate goal for cataract surgery ever since, as it is the equivalent to restorating juvenile visual function. There have been numerous attempts using various kinds of intraocular lens concepts including a refilling of the capsular bag. However, none of these concepts ever fulfilled the high expectations in terms of simplicity of surgery compared to conventional intraocular lenses, prediction of refractive and visual outcome, as well as accommodative amplitude in the long term. The Lumina is different from these concepts as it does not rely on a force transfer via zonular fibers and capsular bag, but moreover it is designed to be directly driven by the ciliary muscle. The integrated spring elements in the haptic move the two lens parts into opposite directions with a maximum amplitude of 0.3 mm, resulting in a relative displacement of the Alvarez surfaces of 0.6 mm and a maximum dioptric change of up to 4 diopters in the spectacle plane.

While there are highly standardized methods for the assessment of refractive predictability/accuracy, visual acuity, etc. the assessment of pseudophakic accommodation is more challenging. When testing for accommodation, recording of all three parameters that define the so called near triad is mandatory: refractive change, vergence, and pupil constriction [41], [42], [43]. The current recommendation for performance testing with accommodative IOLs is to directly measure change in refraction under real conditions, using a real near stimulus, as the mere proximity of the target itself may be a trigger for accommodation [44]. Moreover, literature describes blur and object size as potential cues for accommodation [45], [46]. This excludes defocus curves to confirm accommodation. With defocus curves alone, one could only determine the level of presbyopia correction, however, without revealing the underlying principle, e.g. accommodation, multifocality, extended depth of focus, or monovision. Other methods comprise a direct proof of principle by determining the movement or shape-change during accommodation [44]. This would involve imaging equipment such as OCT or ultrasound biomicrocopy, but inducing accommodation stimuli under such circumstances seems to be impractical. Durkee et al. presented an OCT based system that might override those limitations in the future [47], this will be in the focus of future investigations. The current literature describes several optical setups and devices that have been used to study accommodation. Most of them being experimental setups, only a few that are commercially available. One of them being the open-field autorefractor WAM-5500 (Grand Seiko, Tokyo, Japan) [48], which allowed to record changes in refraction and pupil diameter but not gaze angles. The WAM has been used to determine objective accommodation with the Lumina in previous studies [35]. The device requires readjustment on any change in gaze angles, and small pupil sizes could not be measured, which made dynamic measurements rather impractical. Despite the binocular open field view for the person tested, the WAM-5500 is only capable of measuring a single eye with approx. 5 Hz in the dynamic mode. Therefore, we decided to use the PowerRef3, which allows for measurement of the near triad. The PowerRef’s capability of measuring accommodative reponses has been demonstrated in comparison with the WAM-5500 before [49], [50], [51]. The PowerRef3 is based on the PowerRefractor by Schaeffel, which has been used for numerous studies including myopia and accommodation research [46], [52], [53]. The most important benefits of the PowerRef3 over the WAM-5500 are the simultaneous binocular measurement including pupil diameter and gaze, as well as the higher sampling frequency of up to 50 Hz, the biggest disadvantage is the lack of astigmatism analysis in the dynamic mode, which limits the value of the refractive results. The PowerRef3 has previously been shown its capability to determine dynamic accommodation [51], [53], [54].

Using the PowerRef3 autorefractor we were able to find first proof of pseudophakic accommodation with an accommodative sulcus-based IOL. The amount of accommodation was approx. 2 D at 3 years after implantation, which seems to be sufficient for near work, reading etc. The eye’s accommodative response does not exactly match the accommodative demand, i.e.. the refractive response is lower than expected based on the stimulus distance, which is called accommodative lag. Taking into account that the accommodative lag may be as large as 1 D [55], [56] the total accommodative response of 2 D seen with the Lumina patient fairly explains the good near visual acuity seen clinically (see Table 1, Table 2).

With the three examples given in Fig. 7, Fig. 8, Fig. 9 we demonstrated what we would expect from theory for those patient groups. The young phakic person shows a smooth dynamic change in refraction up to the accommodative demand at near. The presbyopic person shows only little change in refraction, but the remaining components of the near triad work as expected. The pseudophakic person, who iny principle cannot have any accommodation shows only a change in vergence without shift in refraction or pupil size. Wan-Hall and Glasser reported, that the objective accommodative amplitude in pseudophakic eyes is as small as 0.11 ± 0.5 D [57], these values were confirmed by Nemeth et al. with 0.13 ± 0.5 D at a stimulus of 3 D [58]. The three prototype curves (Fig. 7, Fig. 8, Fig. 9) were used to analyze the curve measured with the patient having the Lumina showing a clear and defined change in refraction, pupil reaction and convergence (Fig. 11). The fact that the pupil size showed an increase instead of a decrease may be a proof of principle as it underlines that the change in refraction is not caused by a pupil-dependent EDoF or multifocal power profile of the IOL.

### 4.1 Limitations

A major limitation of the current study is the experimental study design with few selected case reports only. Therefore, additional data and longer follow-ups are required from the clinical study to allow significant conclusions. The small number of cases is mainly attributed to the difficult accommodation measurement with the PowerRef3, which requires an average pupil diameter of more than 4 mm to be maintained throughout the measurement. Most post-cataract patients tend to have a pupil diameter of approximately 3.7 ± 0.8 mm [58], [59], even at low light levels, which prevents the PowerRef3’s algorithm from analyzing the refractive data. Therefore, our measurements were performed at low light levels, as suggested by previous studies on accommodation measurement of pseudophakic eyes [57]. Nevertheless, the low light level is also beneficial in demonstrating that the Lumina is not acting as an EDoF lens, which would provide reading addition only at small pupil diameters. Another limitation with the PowerRef3 is the lack of a distance measurement or linear movement of the test chart in order to (1) have a repeatable measurement, (2) to prevent the patient from viewing off-axis, and (3) to have a diopter scale for the accommodation curve. This would facilitate a linear increase in accommodative demand rather than a constant target speed. A device integrating this was under development by Akkolens but not available for the first measurements presented in this manuscript. Having a diopter-scale would also allow to differentiate between accommodative demand and response. We also found that the PowerRef3 tends to a hyperopic shift, a fact that has also been reported before [49]. The fact that the dynamic mode did not allow for astigmatism measurement is also a major limitation of the PowerRef3. This may be solved in the future with optimized image analysis algorithms and faster computing capabilities. There were other test methods which could have been used to determine accommodative ability, such as placing the stimulus at discrete distances and measuring static refraction or asking the patient to focus from one to another object during measurement. We performed the dynamic measurement to demonstrate the smooth transition from far vision to near vision while the patient is maintaining fixation. Another limitation is the fact, that the working principle of the IOL could only be demonstrated on optical bench testing but not in real patient eyes. This is attributed to the fact that most diagnostic equipment allow only a single eye to be measured while the stimulation of accommodation must be maintained. Furthermore, the required movement of the two lenses is only a few hundred microns, and the OCT picture of the Lumina lacks landmarks that allow determining the displacement of the anterior and posterior lens and the spatial resolution of the currently used OCT is not sufficient. Different OCT systems, such as converted retinal OCTs as presented by Stange et al. [60] have to be investigated whether their resolution allows (a) sufficient penetration depth and (b) better analyses of intraocular lens movement.

### 4.2 Conclusion

The current study shows that the Plusoptix PowerRef3 can be used to determine pseudophakic accommodation after cataract surgery with an accommodative intraocular lens. The accommodative reaction of the Lumina IOL upon a dynamically approaching stimulus was similar to the reaction of a young phakic person, however, with a lower amplitude. Further studies with larger cohort and distance correlated accommodation measurement are required.

## CRediT authorship contribution statement

**Timo Eppig:** Writing – original draft, Visualization, Methodology, Investigation, Formal analysis, Data curation, Conceptualization. **Violetta Müller:** Writing – review & editing, Investigation. **Manuel Seer:** Writing – review & editing, Supervision, Methodology, Data curation. **Alisa Bölke:** Writing – review & editing, Investigation. **Arthur Meßner:** Writing – review & editing, Supervision, Project administration, Funding acquisition. **Michiel C. Rombach:** Supervision, Resources, Investigation, Funding acquisition. **Eckhard Becker:** Writing – review & editing, Investigation. **Jens Schrecker:** Writing – review & editing, Investigation.

## Declaration of competing interest

The authors declare the following financial interests/personal relationships which may be considered as potential competing interests: Michiel Rombach reports a relationship with Akkolens that includes: board membership and equity or stocks. Timo Eppig reports a relationship with AMIPLANT GmbH that includes: employment. Manuel Seer reports a relationship with AMIPLANT GmbH that includes: employment. Arthur Messner reports a relationship with AMIPLANT GmbH that includes: board membership, employment, and equity or stocks. Michiel Rombach has patent Accommodating lens for sulcus plane and capsular bag issued to Akkolens International. Michiel Rombach has patent Method to customize accommodating intraocular lens issued to Akkolens International. If there are other authors, they declare that they have no known competing financial interests or personal relationships that could have appeared to influence the work reported in this paper.
